# Mortality associated with carbapenem-susceptible and Verona Integron-encoded Metallo-β-lactamase-positive *Pseudomonas aeruginosa* bacteremia

**DOI:** 10.1186/s13756-020-0682-4

**Published:** 2020-02-03

**Authors:** Marjolein C. Persoon, Anne F. Voor in’t holt, Cornelia C. H. Wielders, Diederik Gommers, Margreet C. Vos, Juliëtte A. Severin

**Affiliations:** 1 Department of Medical Microbiology and Infectious Diseases, Erasmus MC University Medical Center, Rotterdam, The Netherlands; 2 National Institute for Public Health and the Environment, Bilthoven, The Netherlands; 3 Department of Adult Intensive Care, Erasmus MC University Medical Center, Rotterdam, The Netherlands

**Keywords:** *Pseudomonas aeruginosa*, Mortality, Bacteremia, Anti-bacterial agents, Carbapenemase, Intensive care units, Beta-lactamase VIM

## Abstract

**Background:**

Studies on various Gram-negative bacteria suggest that resistance to carbapenem antibiotics is responsible for increased mortality in patients; however, results are not conclusive. We first assessed the 28-day in-hospital all-cause mortality in patients with Verona Integron-encoded Metallo-β-lactamase-positive *Pseudomonas aeruginosa* (VIM-PA) bacteremia compared to patients with VIM-negative, carbapenem-susceptible *P. aeruginosa* (CS-PA) bacteremia. Second, we identified determinants for mortality and survival.

**Methods:**

All patients with a positive blood culture with VIM-PA or CS-PA between January 2004 and January 2016 were included. Kaplan-Meier survival curves were constructed, and survivors and non-survivors were compared on relevant clinical parameters using univariate analyses, and multivariable analyses using a Cox-proportional hazard model.

**Results:**

In total, 249 patients were included, of which 58 (23.3%) died. Seventeen out of 40 (42.5%) patients with VIM-PA died, compared to 41 out of 209 (19.6%) patients with CS-PA (difference = 22.9%, *P*-value = 0.001). Assumed acquisition of the bacterium at the intensive care unit was significantly associated with mortality (HR = 3.32, 95%CI = 1.60–6.87), and having had adequate antibiotic therapy in days 1–14 after the positive blood culture was identified as a determinant for survival (HR = 0.03, 95%CI = 0.01–0.06). VIM-PA vs CS-PA was not identified as an independent risk factor for mortality.

**Conclusions:**

The crude mortality rate was significantly higher in patients with a VIM-PA bacteremia compared to patients with a CS-PA bacteremia; however, when analyzing the data in a multivariable model this difference was non-significant. Awareness of the presence of *P. aeruginosa* in the hospital environment that may be transmitted to patients and rapid microbiological diagnostics are essential for timely administration of appropriate antibiotics. Acquisition of *P. aeruginosa* should be prevented, independent of resistance profile.

## Introduction

Studies suggest that infections with microorganisms resistant to carbapenem antibiotics may be responsible for increased mortality in patients compared to infections with susceptible microorganisms [1, 2]. However, results are not conclusive and evidence is limited; attributable mortality is therefore still the subject of ongoing investigations [3].

Carbapenem resistance in *Pseudomonas aeruginosa* isolates is an emerging problem, with nosocomial outbreaks of this microorganism occurring worldwide, also in the Netherlands [4–6]. In *P. aeruginosa* isolates, carbapenem resistance may be caused by a variety of mechanisms. However, of particular concern is the production of metallo-beta-lactamase (MBL) enzymes catalyzing the hydrolysis of all classes of beta-lactam antibiotics except monobactams [7]. Currently, the Verona Integron-encoded MBL (VIM) is the most widespread MBL in *P. aeruginosa*, with VIM-2 being the major source of worldwide outbreaks [8]. Infections (e.g. bacteremia) with VIM-positive *P. aeruginosa* (VIM-PA) are mainly seen in patients with a suppressed immune system or otherwise compromised, for example in patients admitted to the intensive care unit (ICU) [9]. Published mortality rates for bloodstream infections with *P. aeruginosa* are between 20 and 50%, with the rate of carbapenem-resistant *P. aeruginosa*-attributable deaths among those patients being between 8 and 18% [2, 10]. However, the question still remains if mortality in these patients is high because of 1) inadequate or delayed appropriate antibiotic therapy, 2) severity of underlying disease, or 3) because of the VIM gene and the subsequent carbapenem resistance [1, 9, 11, 12]. Moreover, clones with and without carbapenemase genes can also differ in virulence characteristics, which could lead to differences in mortality rates between patients with different clones; independent of the resistant mechanism [13].

First, we aimed first to compare the 28-day in-hospital all-cause mortality in patients with a VIM-PA bacteremia to patients with a bacteremia with VIM-negative, carbapenem-susceptible *P. aeruginosa* (CS-PA) in a large tertiary hospital in the Netherlands. Second, we aimed to identify risk factors for mortality by *P. aeruginosa* bacteremia in this setting.

## Methods

### Ethics statement

Written approval to conduct this study was received from the medical ethical research committee from the Erasmus MC University Medical Center (Erasmus MC), Rotterdam, the Netherlands (MEC-2015-306).

### Setting

This study was conducted at the Erasmus MC. During the study period, this was a 1200-bed university hospital organized into 48 different wards [14]. Of these wards, three were high-level adult ICUs, and each had only single-occupancy rooms. Since 2008 ICU patients received selective digestive tract decontamination if expected to be on mechanical ventilation for > 48 h or anticipated to be admitted to the ICU for > 72 h [15]. The number of clinical admissions and clinical admission days for the study period are described in a previous publication [5].

### Patient inclusion and data collection

Patients were included if identified with a positive blood culture with *P. aeruginosa* between January 1st 2004 and January 1st 2016. Only the first isolate of each patient was included. We excluded the following patients: (i) patients < 18 years old, (ii) non-hospitalized patients, (iii) if the first positive blood culture contained more than one pathogenic microorganism, and (iv) patients identified with a carbapenem-resistant *bla*_VIM_-negative *P. aeruginosa* isolate. Positive blood cultures with both *P. aeruginosa* and coagulase-negative staphylococci (CoNS) were included when the CoNS were considered to be contamination. These cultures were excluded if the CoNS was cultured in a second blood culture and if the CoNS was considered clinically relevant (e.g. antibiotic treatment was started or other actions were performed).

We collected the following patient data from the electronic patient records: (i) age at day of first positive blood culture with *P. aeruginosa*, (ii) sex, (iii) date of first positive blood culture with *P. aeruginosa* and susceptibility pattern, (iv) department of acquisition, defined as the department at which the patient was admitted 48 h before the positive blood culture: if the patient was positive at admittance this was scored as ‘at home’ or ‘at other healthcare facility’, (v) date of admission to and date of discharge from our hospital, (vi) death 28 days after first positive blood culture yes/no and date of death, (vii) nosocomial infection, defined as a positive blood culture with *P. aeruginosa* > 48 h after admission, (viii) neutropenia, defined as an absolute neutrophil count of < 0.5 X10^9^ cells/L during presentation of bacteremia; neutrophil count was assessed 2 days before the first positive blood culture was taken to 7 days afterwards, (ix) use of corticosteroids 28 days before until 28 days after the positive blood culture with *P. aeruginosa*, (x) use of other immunosuppressive agents than corticosteroids in 28 days before until 28 days after positive blood culture with *P. aeruginosa*, and (xi) antibiotic use. During analyses, viii, ix and x were combined and reported as being immunocompromised yes or no.

Use of one or more of the following antibiotics was checked from the day the first positive blood culture was taken to 14 days thereafter: piperacillin/tazobactam, gentamicin, tobramycin, ceftazidime, ciprofloxacin, colistin and carbapenems. These antibiotics have antipseudomonal activity and are part of our local antibiotic policy. Piperacillin without tazobactam is not used in our hospital. Three definitions of adequate antibiotic use were applied. Antibiotic use at the moment the first positive blood culture was drawn was defined as adequate when administration of at least one antibiotic agent for which the *P. aeruginosa* isolate is susceptible was given for at least 24 h (adequate antibiotic use 1). In the 2 weeks after the blood culture was drawn, antibiotic use was defined as adequate when at least one administered antibiotic agent for which the *P. aeruginosa* isolate is susceptible was administered for at least 24 h (adequate antibiotic use 2). Patients who deceased within 24 h after the first positive blood culture were excluded from analyses with variable adequate antibiotic use 2. As third definition, we combined adequate antibiotic use 1 and adequate antibiotic use 2 (adequate antibiotic use total).

For all patients, we calculated the Charlson comorbidity score at admission and for patients admitted to the ICU we calculated the Acute Physiology And Chronic Health Evaluation (APACHE) score within 24 h after admission to the ICU [16, 17]. The Charlson comorbidity score is a method used to predict mortality by assigning different weights to comorbidities [17]. We used an updated Charlson comorbidity index which was validated and published by Quan et al. in 2011 [17].

The primary endpoint of this study was 28-day in-hospital all-cause mortality. Patients were followed until 1) in-hospital death up until 28 days after the first positive blood culture, 2) hospital discharge within 28 days after the first positive blood culture, or 3) until day 28 if still admitted by that time. Readmissions within 28 days after the first positive blood culture were also considered and data was used for analyses.

### Microbiological methods

Blood cultures taken on clinical indication were processed in the laboratory using standard microbiological methods (BACTEC system BD). Identification and susceptibility testing of Gram-negative aerobic bacilli were performed using Vitek2 (bioMérieux, Marcy l’Etoile, France). Since January 2013, the MALDI-TOF (Bruker Daltonics, Bremen, Germany) was used for identification. Breakpoints were in accordance to Clinical and Laboratory Standards Institute (CLSI) guidelines until August 27th 2013, thereafter European Committee on Antimicrobial Susceptibility Testing (EUCAST) guidelines were used. In case of suspected growth of carbapenemase-producing *P. aeruginosa* or multidrug-resistant *P. aeruginosa*, an in-house polymerase chain reaction (PCR) for detection of *bla*_VIM_ on LightCycler 480 (Roche Diagnostics, Almere, The Netherlands) was performed as previously described [18, 19].

### Statistical analysis

To calculate differences in mortality, crude mortality of patients with CS-PA was subtracted from crude mortality of patients with VIM-PA. Kaplan-Meier survival curves were constructed for these 2 groups for 28-day in-hospital survival. The log rank test was performed to statistically compare the two curves. Univariate analyses in order to compare survivors to non-survivors were conducted using a *t* test, the independent samples median test or the Mann-Whitney U test when appropriate. All analyses were performed using SPSS version 24 (IBM Corp., Armonk, New York, USA). Concerning the multivariable analysis: because some patients were discharged and readmitted within 28 days, a start-stop-event Cox-proportional hazard model was fitted using the R project for statistical computing, version 3.4.3 (Vienna, Austria) to calculate hazard ratios (HRs) for 28-day in-hospital mortality. The selection of variables in the multivariable model was based on clinical relevance and results of the univariate analysis, with age and sex as standard parameters included. To test if the model improved when relevant interaction terms were added, models were compared using the ANOVA statistic (analysis of deviance). *P*-values of < 0.05 were considered statistically significant, and a 95% confidence interval (CI) was used.

## Results

### Patient inclusion and characteristics

**Fig. 1:**
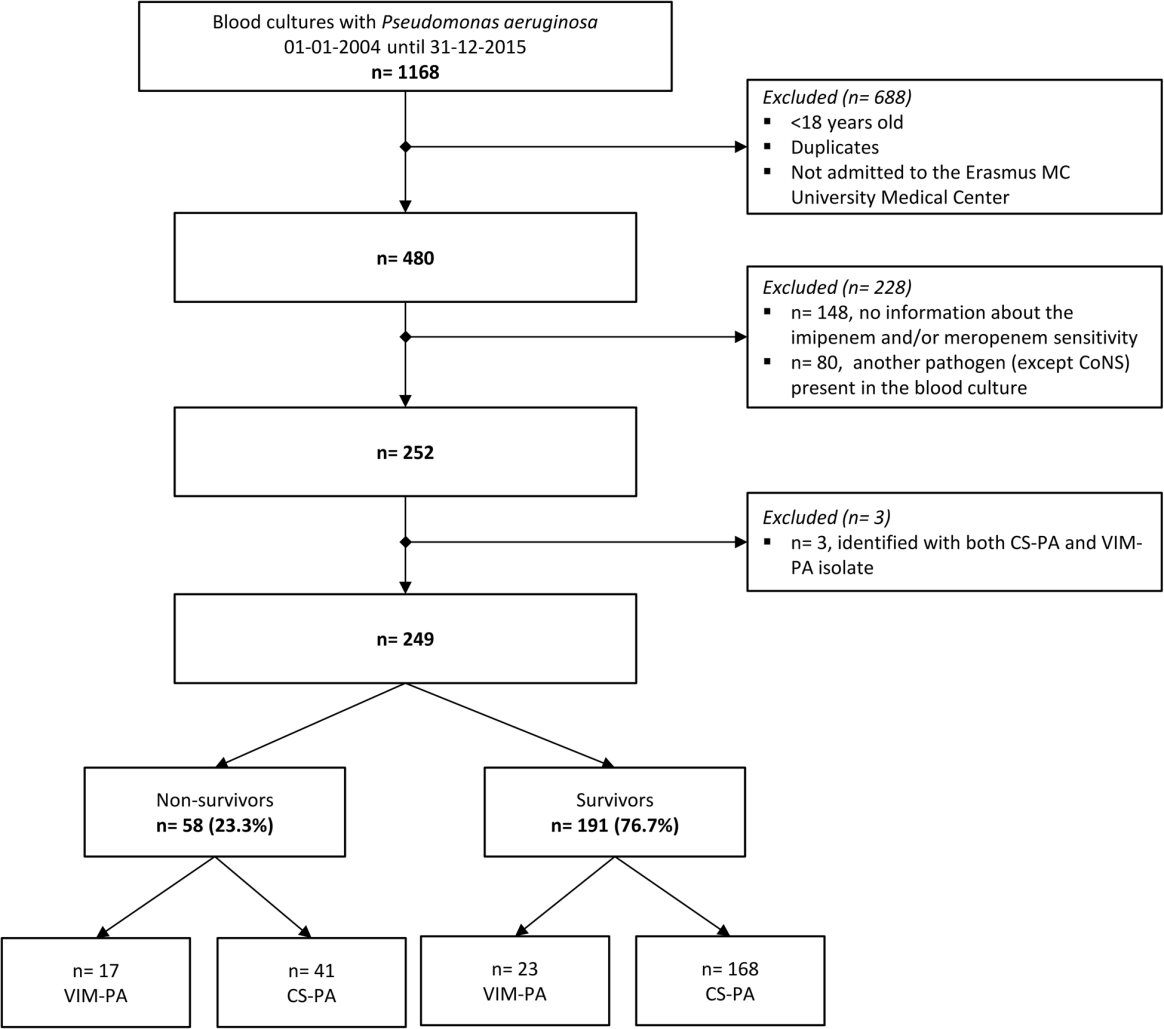
Flow diagram of patient inclusion. Abbreviations: CoNS, coagulase-negative staphylococci; VIM-PA, Verona Integron-encoded Metallo-β-lactamase-positive *Pseudomonas aeruginosa*; CS-PA, VIM-negative, carbapenem-susceptible *Pseudomonas aeruginosa*

Between 2004 and 2016, 249 patients with a *P. aeruginosa* positive blood culture were included in this study (Fig. 1), of which 40 (16.1%) were identified with VIM-PA, and 209 (83.9%) with CS-PA. In blood cultures of three patients, a CoNS was present in a single blood culture, which was considered to be contamination (i.e. *n* = 1 *Staphylococcus epidermidis*, n = 1 *S. hominis*, and *n* = 1 *Staphylococcus* species). In another patient, a CoNS was found in a second blood culture together with a *P. aeruginosa*, however, the two blood cultures taken on the same day were negative, as well as a blood culture taken a few days later. Furthermore, no antibiotics were started for this CoNS. Therefore, this was also considered to be contamination. Overall, 159 male (63.9%), and 90 (36.1%) female patients were included, with a mean age of 59.5 years old. Regarding age and sex, there were only small, non-significant differences between survivors and non-survivors (Table 1). Twenty-eight patients (11.2%) died within 48 h after the positive blood culture, 11 with a VIM-PA bacteremia (4.4% total, 27.5% of patients identified with VIM-PA), and 17 with a CS-PA bacteremia (6.8% total, 8.1% of patients identified with CS-PA) (*P*-value < 0.001). Of these patients, 4 with a VIM-PA bacteremia (1.6% total, 10.0% of patients identified with VIM-PA), and also 4 patients with a CS-PA bacteremia (1.6% total, 1.9% of patients identified with CS-PA) died within 24 h (*P*-value = 0.008). For these 8 patients, analysis on adequate antibiotic use 2 could not be performed.

**Table 1:** Patient-related clinical variables of survivors and non-survivors

Characteristic	Non-survivors (*n* = 58)	Survivors (*n* = 191)	*P*-value
Male gender (%)	38 (65.5)	121 (63.4)	0.764
Mean age ± SD at time of first blood culture with PA	60.5 ± 12.9	59.3 ± 14.1	0.539
Nosocomial infection (%)	45 (77.6)	113 (59.2)	0.011
VIM-PA bacteremia (%)	17 (29.3)	23 (12.0)	0.002
Nosocomial infection (%)	17 (100)	19 (82.6)	0.070
Adequate AB therapy 1 (%)^a^	5 (31.3)	8 (42.1)	0.818
Adequate AB therapy 2 (%)^b^	5 (38.5)	23 (100)	< 0.001
Median Charlson score(range)^c,d^	2 (0–6)	3 (0–10)	0.639
Ward of acquisition; ICU (%)	29 (50)	32 (16.8)	< 0.001
Median APACHE score (range)^e^	22 (10–43)	22.5 (7–41)	0.757
APACHE > 25 (%)^e^	6 (11.8)	7 (35.0)	0.588
Immunocompromised^f^	29 (50.0)	84 (44.0)	0.420
Adequate AB therapy total (%)^g,h^	31 (63.3)	157 (98.1)	< 0.001
Adequate AB therapy 1 (%)^i^	17 (30.9)	52 (27.8)	0.654
Adequate AB therapy 2 (%)	26 (52.0)^h^	156 (97.5)^j^	< 0.001

Abbreviations: *SD* Standard deviation, *PA Pseudomonas aeruginosa, VIM-PA* Verona Integron-encoded Metallo-β-lactamase-positive *Pseudomonas aeruginosa, APACHE* Acute Physiology and Chronic Health Evaluation, *ICU* Intensive care unit, *AB* Antibiotic; adequate AB therapy 1, day 0 for at least 24 h adequate AB use; adequate AB therapy 2, days 1–14 adequate AB use, for at least 24 h. Bold *P*-values are significant

^a^non-survivors = 1 missing. ^b^Four patients were excluded because they deceased within 24 h. ^c^Quan et al. [17]. ^d^Charlson: survivors = 1 missing, non-survivors = 1 missing. ^e^APACHE: survivors = 12 missing, non-survivors = 7 missing. ^f^Combination of variables neutropenia; use of corticosteroids and use of immunosuppressive agents other than corticosteroids. ^g^Adequate AB total: survivors = 31 missing, non-survivors = 1 missing. ^h^Eight patients from the non-survivors were excluded because they deceased within 24 h. ^i^Adequate AB 1: survivors = 4 missing, non-survivors = 3 missing. ^j^Adequate AB 2: survivors = 31 missing

### 28-day in-hospital all-cause mortality

**Fig. 2:**
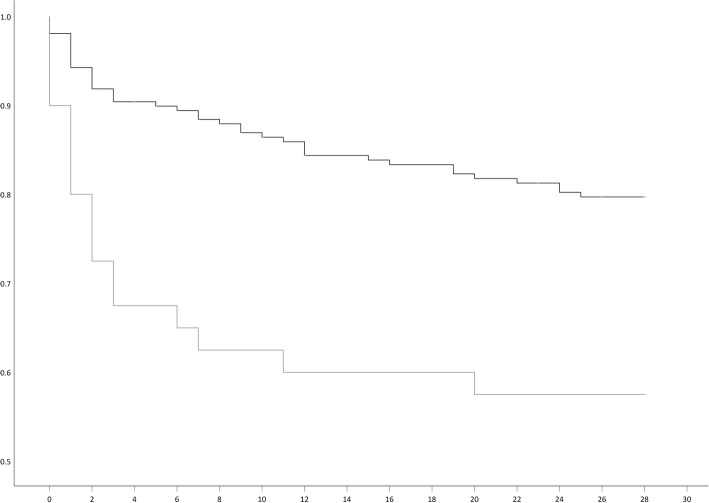
Kaplan Meier curve of patients with VIM-negative, carbapenem-susceptible *Pseudomonas aeruginosa* (dark-grey line) and patients with VIM-positive *Pseudomonas aeruginosa* (light grey line). Y-axis, cumulative survival, X-axis, days. Log Rank *P-* value = 0.001

Fifty-eight (23.3%) out of 249 patients died within 28 days in the Erasmus MC, and 191 patients (76.7%) survived during follow-up. Seventeen out of 40 (42.5%) patients with VIM-PA died within 28 days in the Erasmus MC, compared to 41 out of 209 (19.6%) patients with CS-PA. Therefore, the difference in crude mortality rate was 22.9%. Additionally, the Kaplan-Meier survival curve visualizes the difference between patients identified with VIM-PA compared to CS-PA over time (Log Rank *P-* value = 0.001) (Fig. 2). Patients with VIM-PA deceased shortly after the positive blood culture (median = 2 days, range 0–20). This was sooner compared to patients with CS-PA (median = 5 days, range 0–25). Univariate analyses showed that mortality was associated with a nosocomial infection, having VIM-PA instead of CS-PA, acquisition of the bacterium in the ICU, and inadequate antibiotic therapy in total and at days 1–15 after the positive blood culture (adequate AB therapy 2) (Table 1). The Charlson score was not associated with mortality, as was the APACHE score on admission to the ICU (Table 1). Between patients identified with a VIM-PA or CS-PA there were no significant differences regarding the Charlson or the APACHE score.

**Fig. 3:**
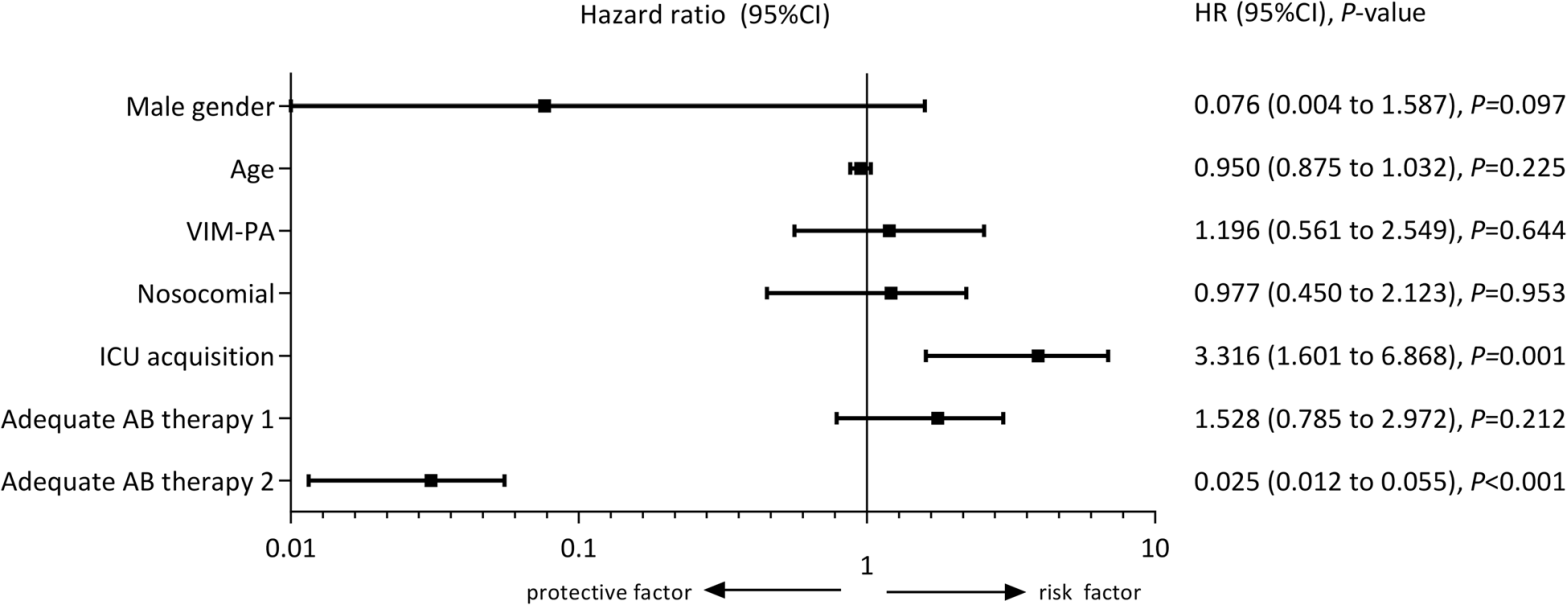
Multivariable analysis to identify determinants significantly related to mortality after *Pseudomonas aeruginosa* bacteremia. Abbreviations: ICU, intensive care unit; AB, antibiotic; HR, hazard ratio; 95%CI, 95% confidence interval; VIM-PA, Verona Integron-encoded Metallo-β-lactamase-positive *Pseudomonas aeruginosa;* adequate AB therapy 1, day 0 for at least 24 h adequate AB use; adequate AB therapy 2, days 1–14 adequate AB use, for at least 24 h

In the multivariable model, the following variables were included: (i) sex, (ii) age, (iii) VIM-PA vs. CS-PA, (iv) ICU as ward of acquisition, (v) nosocomial acquisition of *P. aeruginosa*, (vi) adequate antibiotic use 1 and (vii) adequate antibiotic use 2. Figure 3 shows the results of the final multivariable model, in which acquisition in the ICU was identified as a statistically significant risk factor for mortality in all patients (HR = 3.32, 95%CI = 1.60 to 6.87). Having had adequate antibiotic therapy in days 1–14 after the positive blood culture (adequate antibiotic use 2) was identified as a determinant for survival (HR = 0.03, 95%CI = 0.01 to 0.06) (Fig. 3). Although significantly associated in univariate analyses, VIM-PA vs. CS-PA was not identified as an independent risk factor for mortality, thus after correcting for all other variables present in the model.

## Discussion

The 28-day in-hospital all-cause mortality in patients with a bacteremia with VIM-PA was significantly higher compared to patients with a bacteremia with CS-PA, with a difference in crude mortality rate of more than 22%. In the systematic review published by Zhang et al. the attributable mortality ranged from 8 to 18.4%, with 4 studies included from Brazil, Spain, Korea and the U.S. [2]. Thus, in our center in the Netherlands the rate is high. Despite the difference in crude mortality between VIM-PA and CS-PA, in the multivariable model VIM-PA was not identified to be significantly associated with mortality. This was also reported by Peña et al. They described that the effect of resistance on mortality decreased with higher Charlson scores, i.e. the effect disappeared in the presence of comorbidities [1]. We did identify that acquisition of *P. aeruginosa* in the ICU was significantly associated with mortality, which could be related to disease severity. However, in the univariate analysis we did not identify any differences between survivors and non-survivors, and between patients with VIM-PA and CS-PA regarding the Charlson score at hospital admission or APACHE score on admission to the ICU. The Charlson comorbidity score is obtained with data from time of admission and may therefore not represent the severity of disease during admission, especially not in a tertiary care hospital where patients need and receive high level care; this includes broad-spectrum antibiotics, several medical devices (e.g. mechanical ventilation, central venous catheters) and close monitoring by physicians and nurses. This makes these patients more vulnerable for acquisition and infection by microorganisms, which could lead to mortality. Furthermore, acquisition of *P. aeruginosa* in the hospital could be responsible for deterioration of the patients’ clinical condition.

Patients with VIM-PA deceased shortly after the positive blood culture, which was sooner compared to patients with CS-PA. A possible explanation could be that more than half of patients with VIM-PA acquired this bacterium in the ICU, compared to 17.2% of patients with CS-PA. Acquisition in the ICU may therefore be an indication of increased severity of disease at the moment of acquisition. A second possible explanation could be differences in receiving timely adequate antibiotic therapy. Adequate antibiotic use 2 (i.e. days 1–14 after the positive blood culture for at least 24 h) was identified as a protective factor, which means that this decreased risk of mortality. Survivors were two times more likely to receive correct antibiotic therapy than non-survivors (97.5% vs. 52.0%, respectively). This is also described by González et al., Paulsson et al., DiMondi et al., Peña et al. and Raman et al. [20–24]. This big difference and very low percentage of correct antibiotic use in non-survivors could be largely explained by the time of death: 78% of patients died between 24 and 48 h after the first positive blood culture. In some cases, treatment with for example meropenem was started, however not given for > 24 h before the patient died. In other cases, an incorrect antibiotic was given e.g. for which the identified *P. aeruginosa* was resistant, or no antibiotics were started. Since VIM-PA in our patient group was found to be resistant for several antibiotic classes, it can be hypothesized that patients with a VIM-PA bacteremia have an increased risk of receiving inadequate empirical antibiotic therapy. However, in our patient group there was no difference in receiving adequate therapy 1, 2 or total between patients with VIM-PA and CS-PA. A possible explanation is that most patients with VIM-PA were in the ICU when having a VIM-PA bacteremia, and in 52% VIM-PA was acquired in the ICU. At the ICU, patients are monitored very closely and screening cultures of various sample-sites are taken regularly. Therefore, colonization of VIM-PA in a patient may be detected before infection occurs, facilitating starting adequate therapy when indicated. When comparing patients with acquisition of *P. aeruginosa* in the ICU to patients with acquisition in non-ICU wards, there was a difference in receiving adequate antibiotic therapy 1 (ICU, 36.7%; non-ICU, 25.8%), however this was not statistically significant (*P-* value = 0.107).

### Limitations and strengths

Our study has some limitations. First, patient groups with VIM-PA and CS-PA were not matched. Ideally, matching should have been done on life-expectancy and/or severity of illness on the day of positive blood culture. Second, this is a retrospective study conducted in a single tertiary care hospital in the Netherlands; therefore, the results may not be generalizable to other institutions and/or countries. However, we feel that a single center study design is preferred above a multi-center when studying the sole effect of VIM presence above other patient- and care-related risk factors, as the latter differ considerably between centers. Third, because of the low total number of deceased patients (*n* = 58), small but true differences may have been missed.

A strength of our study is that despite it being a single center study, a large group of patients could be included. Second, we focused on solely the VIM gene.

### Conclusions and implications

The crude mortality rate was significantly higher in patients with a VIM-PA bacteremia compared to patients with a CS-PA bacteremia in our university hospital. Acquisition of VIM-PA and CS-PA mainly occurred in the ICU in a vulnerable ICU patient group, 51.7 and 31.3% respectively. Additionally, patients deceased rapidly after acquiring *Pseudomonas aeruginosa.* Despite the higher crude mortality rate for VIM-PA, our study showed that in our population bacteremia with CS-PA were equally important regarding clinical outcome compared to VIM-PA, since multivariable analyses showed no difference between these two groups. Infections with CS-PA should therefore not be underestimated. Adequate antibiotic therapy for VIM-PA as well as for CS-PA has shown to be a determinant for surviving. Therefore, since VIM-PA are resistant to many antibiotic groups and consequently more difficult to treat, awareness of the presence of VIM-PA in the hospital environment and rapid microbiological diagnostics and sensitivity analysis are essential for timely administration of adequate antibiotics. Acquisition of *P. aeruginosa* should be avoided and prevented, in particular in the ICU, independent of resistance profile. To elucidate the sole role of VIM we propose to perform a multi-center study in different countries with a design matched on life expectancy.

## Data Availability

The datasets generated and analyzed during the current study are not publicly available due to privacy regulations but are available from the corresponding author on reasonable request.

## Abbreviations

APACHE: The Acute Physiology And Chronic Health Evaluation score
CI: Confidence interval
CLSI: Clinical and Laboratory Standards Institute
CoNS: Coagulase-negative staphylococci
CS-PA: Carbapenem-susceptible *P. aeruginosa*
Erasmus MC: Erasmus MC University Medical Center
EUCAST: European Committee on Antimicrobial Susceptibility Testing
ICU: Intensive care unit
MBL: Metallo-beta-lactamases
PCR: Polymerase chain reaction
VIM: Verona Integron-encoded MBL
VIM-PA: VIM-positive *P. aeruginosa*
